# Are British urban foxes (*Vulpes vulpes*) “bold”? The importance of understanding human–wildlife interactions in urban areas

**DOI:** 10.1002/ece3.7087

**Published:** 2020-12-26

**Authors:** Roberto Padovani, Zhuoyu Shi, Stephen Harris

**Affiliations:** ^1^ School of Biological Sciences University of Bristol Bristol UK

**Keywords:** boldness, management, neophobia, risk‐taking, social status, wariness

## Abstract

Human–wildlife interactions are believed to be increasing in urban areas. In Britain, numerous media reports have stated that urban foxes (*Vulpes vulpes*) are becoming “bolder,” thereby posing a risk to public safety. However, such claims overlook how an individual's personality might influence urban fox behavior. Personality determines multiple aspects of an animal's interactions with both conspecifics and its environment, and can have a significant impact on how people perceive wildlife. Furthermore, describing urban foxes as “bold” confounds two different but inter‐related behaviors, both of which influence an animal's propensity to take risks. Neophobia affects an animal's reaction to novelty, wariness its reaction to potential threats. Since urban wildlife frequently encounters both novel and threatening stimuli, a highly adaptable species such as the red fox might be predicted to exhibit reduced neophobia and wariness. We investigated how social status influenced both behaviors in Bristol's fox population. Dominant foxes were significantly more neophobic and warier than subordinates, which adopt a more exploratory and risk‐taking lifestyle to meet their energetic and other needs. We found no seasonal effect on neophobia and wariness, although this may be due to sample size. The presence of conspecifics decreased neophobia for dominants, and wariness for both dominants and subordinates. We highlight the importance of considering animal social status and personality when planning management protocols, since interventions that destabilize fox social groups are likely to increase the number of subordinate foxes in the population, thereby increasing rather than decreasing the number of interactions between humans and urban foxes.

## Graphical abstract

There is considerable public interest in the behavior of urban foxes, and whether “bold” foxes pose a risk to public safety. We used experiments on free‐living foxes to examine how personality and social status influence the propensity of urban foxes to take risks. We show that subordinate foxes have to adopt a more exploratory and risk‐taking lifestyle than dominants in order to meet their energetic and other needs.

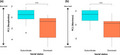

## 1 INTRODUCTION

There is a widespread perception that interactions between people and wildlife are increasing as human populations and urban areas expand. This is of particular concern with carnivores (Curtis & Hadidian, 2010; Elliot et al., 2016; Knopff et al., 2016), since even small species can be perceived as threatening (König, 2008). Several canids have adapted to coexist with humans: in particular, coyotes (*Canis latrans*) are widespread in urban areas in North America and red foxes (*Vulpes vulpes*) in Europe and Australia (Gehrt, 2004; Gehrt & Riley, 2010; Soulsbury et al., 2010).

Red foxes colonized most British cities over 80 years ago (Harris & Rayner, 1986a), and urban fox population densities can be several times higher than in rural areas (Baker et al., 2001; Harris & Rayner, 1986b). At high densities, foxes live in social groups consisting of up to ten adults (i.e., animals >1 year old), with equal numbers of adult males and females; there is a dominant pair and a variable number of subordinates (Dorning & Harris, 2019a). In Britain, urban foxes frequently live and breed in residential back gardens, where they have a diversity of interactions with people, many of which have been characterized as “conflict” (e.g., Soulsbury & White, 2015). However, conflict implies repeated and conscious antagonism between wildlife and humans, that is, it is a two‐way process (Davidar, 2018), whereas most negative interactions between people and urban foxes are minor and best described as nuisance (Baker & Harris, 2007). The misuse of the term “conflict” masks the underlying complexities of human–wildlife interactions, hinders management, and has an adverse impact on how we understand and investigate these relationships (Hill, 2018; Peterson et al., 2010). In this paper, we use the term “interactions” because this better reflects the relationships between foxes and people in British cities.

**FIGURE 1:**
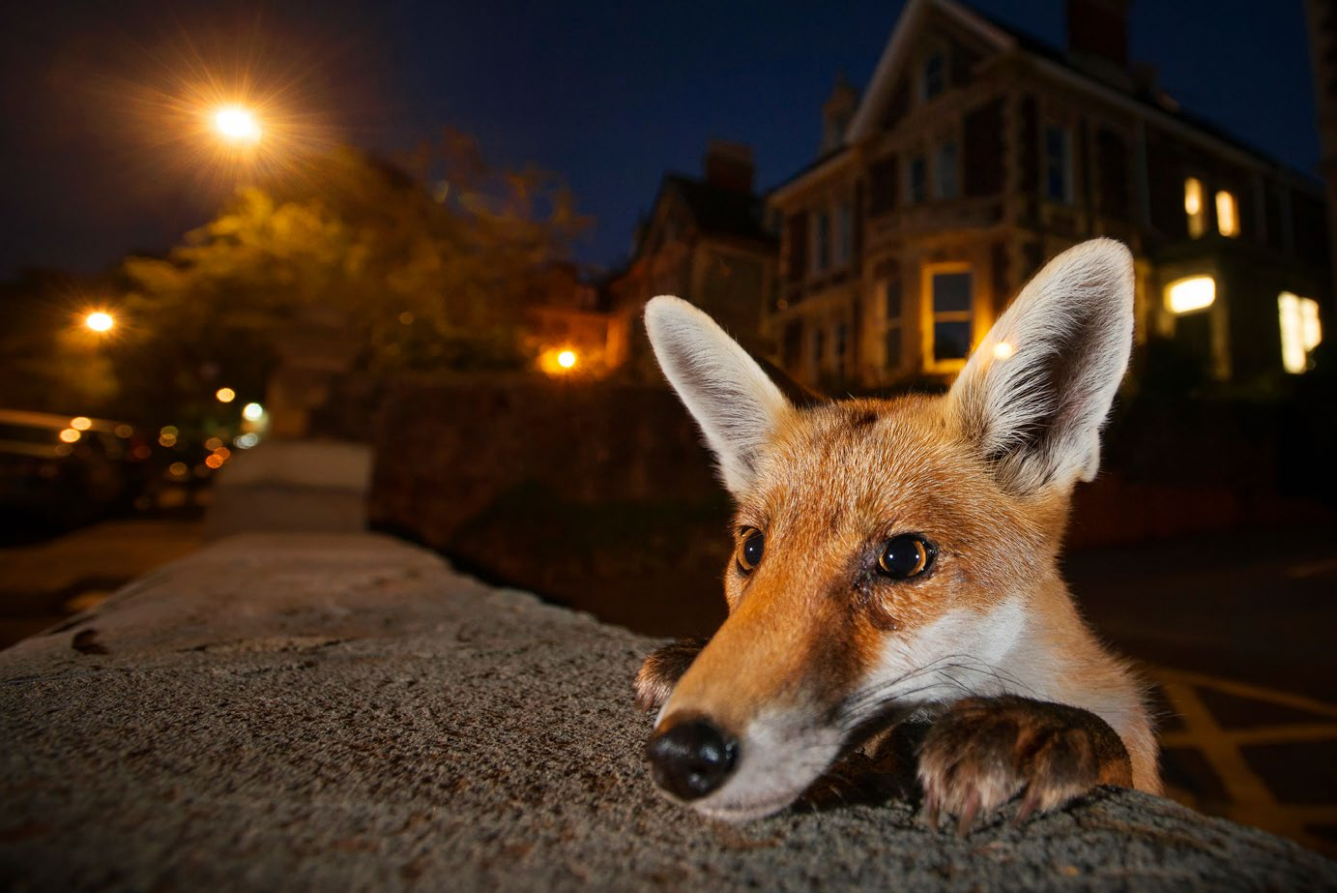
An urban fox frequently observed on the University of Bristol's campus, where it would approach people to solicit food during the day as well as at night. Photograph copyright Sam Hobson

For more than a decade, numerous articles in the British press have claimed that urban foxes are becoming “bolder” (Cassidy & Mills, 2012). Characteristics attributed to bold foxes include being more willing to enter houses and approach and develop bonds with people (Figure 1), such as being hand‐fed. These animals are often described in the press as “cunning,” “daring,” “fearless,” and “sly” and are said to pose a particular risk to babies and young children (e.g., Crowden, 2013; Gray, 2009; Lindsay‐Smith, 2011), but see Bridge and Harris (2020). This perception has been enhanced by claims that bolder foxes “are more likely to be more successful in obtaining food” in urban areas, which is leading to selection for “more courageous foxes,” and that foxes that are less bold “are unlikely to thrive in cities” (Scott, 2017).

However, such assertions fail to recognize the importance of personality in animals (Dall et al., 2004). Personality is exhibited as consistent differences in behavior between individuals in a population when faced with the same environmental or social stimulus (Dingemanse & Réale, 2005), and boldness, activity, and/or aggressiveness are positively related to life‐history traits in a range of taxa (Biro & Stamps, 2008). Different behaviors emerging within the same species or population reduce intraspecific competition by enabling individuals to occupy diverse niches and thereby exploit different resources (Stamps & Groothuis, 2010).

Novel stimuli, such as food items, objects, or places, may elicit attraction (neophilia), repulsion (neophobia), or indifference (Travaini et al., 2013), with less explorative individuals displaying neophobia, typically exhibited by hesitation, avoidance, or caution (Harris & Knowlton, 2001). The degree of neophobia exhibited by an individual in any given situation is believed to depend on the trade‐off between the potential risks and benefits of exploring the novel stimulus (Lima & Dill, 1990). It might be expected that neophobia would decrease in species that exploit urban areas, as a result of heightened exposure, and consequent habituation, to novel stimuli. However, there is evidence for both decreased (Lowry et al., 2013) and increased neophobia in urban‐living species (Miranda et al., 2013).

While an animal's propensity to take risks is often referred to as boldness (Mettler & Shivik, 2007; Wilson et al., 1994), the term specifically describes an individual's behavior in response to potentially threatening situations that have been experienced previously (Réale et al., 2007). Since boldness, defined in this sense, and neophobia both stem from an individual's internal state of risk perception, an individual that is more neophobic around novel stimuli may also be more wary when faced with potential threats (Darrow & Shivik, 2009; Séquin et al., 2003). There may also be seasonal effects on an animal's willingness to take risks. For instance, adult foxes cross roads more often during the spring and summer while provisioning cubs (Baker et al., 2007), and adult female mortality is higher in May, when they are cub provisioning, than in all other months except January, which is the peak of the mating season (Harris & Smith, 1987).

In this paper, we use the term neophobia to indicate an animal's reaction to novelty, wariness to describe caution of, and aversion to, potentially threatening stimuli, and boldness to describe risk‐taking behavior generally. We investigated neophobia and wariness as related but separate traits and examined status‐specific and seasonal differences in neophobia and wariness in urban foxes. Additionally, we discuss whether there is any evidence of selection for increased boldness in urban foxes, and how social status might influence public perceptions of, and attitudes toward, foxes. We used experiments on free‐living foxes to test four predictions: (aa) social status will influence a fox's response to novel stimuli; (b) social status will influence a fox's response to threatening stimuli; (c) season will affect a fox's response to novel and threatening stimuli; and (d) the presence of conspecifics will affect a fox's response to novel and threatening stimuli. We use these data to help understand human–fox interactions in urban areas.

## 2 METHODS

### 2.1 Study area and experimental design

**FIGURE 2:**
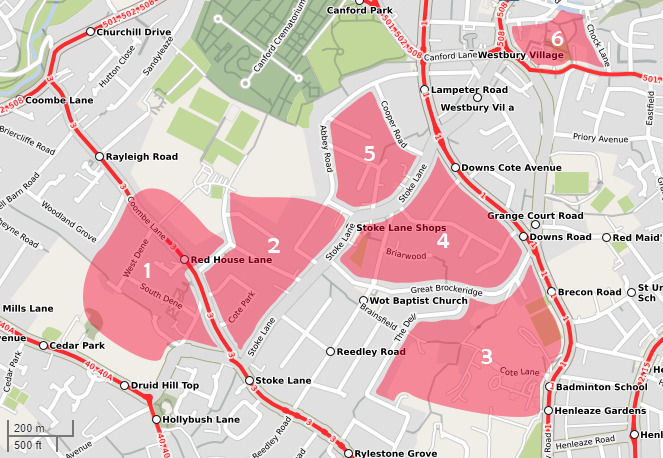
Territories of the six fox social groups in north‐west Bristol used in the study. The territorial boundaries were established from radio‐tracking and camera‐trapping data, and physical boundaries such as roads. Base map courtesy of https://www.openstreetmap.org

We studied six urban fox groups in north‐west Bristol, UK (Figure 2), which is the site of an intensive 40‐year study into the behavior and ecology of urban red foxes (Baker et al., 1998, 2000, 2001, 2004; Baker, Newman, et al., 2001; Iossa et al., 2009). The study area consisted predominantly of semi‐detached housing built in the 1930s; human density was 30 people/ha. We used radio‐tracking, camera‐trapping, and physical boundaries such as roads (Saunders et al., 1993) to identify territory boundaries; average fox territory size was 0.12 km^2^. To avoid testing the same animals at different sites, we only used one garden in each territory for our experiments and selected rear gardens where (a) the foxes were already being fed by the householders to maximize visitation rates, (b) there was a suitable lawn for the study, and (c) there was only one entry point to ensure that the foxes always approached from the same direction.

**FIGURE 3:**
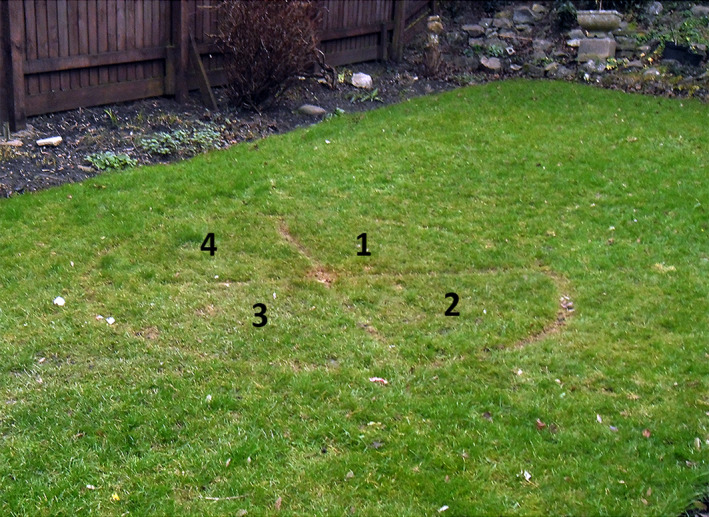
The experimental arena marked out on a lawn with cuts 4‐cm wide, producing small differences in grass height: This was considered to be the least invasive method of marking the arena. The numbers denote the four quadrants; quadrant 1 was closest to the entry point, and quadrant 3 was closest to the house

**FIGURE 4:**
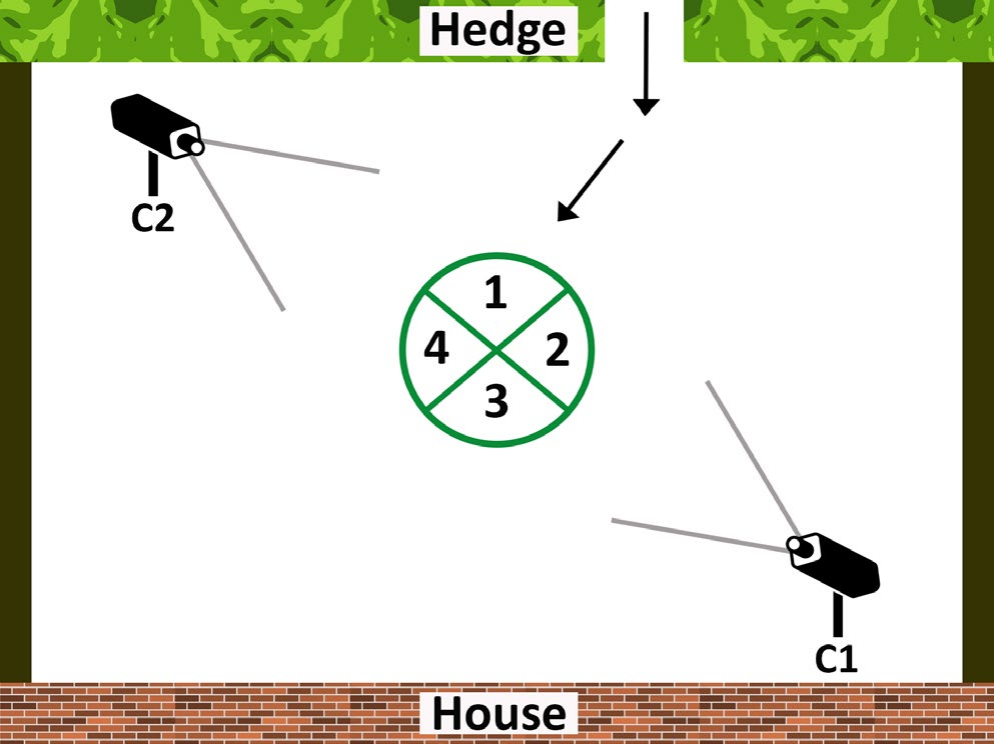
Schematic of a typical back garden used in the study, showing the location of the experimental arena and four quadrants, direction of approach of the fox from a single access point, and the location and angles of view of the two video cameras. Not to scale

In each garden, we set up an experimental arena consisting of a circle 2 m in diameter, divided into quadrants (Travaini et al., 2013): The arena was cut into the lawn with a small pair of scissors (Figure 3). The 4‐cm‐wide cut marks were visible on video recordings day and night but, since urban foxes encounter frequent changes in their environment, including cutting lawns, slight differences in grass height were unlikely to influence their behavior: foxes sometimes explored the circle on the day it was cut but ignored it thereafter. Quadrant 1 was closest to the point of entry of the foxes, quadrant 3 was closest to the house, and quadrants 2 and 4 faced to the sides of the garden (Figure 4). Since quadrants 2 and 4 were equivalent in terms of their position, we only tested the behavior of foxes in quadrant 2.

We used two 20‐day experimental periods, in late November/early December 2014 and May 2015, to examine seasonal influences. Late November/early December is the peak dispersal period for red foxes (Harris & Trewhella, 1988), and May is when most adults in a social group help provision young cubs (Baker et al., 1998); these changes in behavior are associated with seasonal differences in intergroup movements and social relationships (Dorning & Harris, 2019b, 2019c). Any cubs recorded in May were excluded from the analyses. Experiments were started a week after the arena was cut. Food was placed in the center of the arena every evening between 17:00 and 20:00 (GMT in November/December, BST in May). The type of food varied between households but was consistent in both type and amount within each garden. Some households fed the foxes on most days; those that provided food less frequently began feeding the foxes every night for 10 days prior to the first experimental day to encourage regular visits by the foxes.

The arena was recorded continuously for each 20‐day period using two CCTV cameras, each attached to a 2‐m long wooden pole and linked to a digital video recorder (Home Guard DIY CCTV kit, Storage Options). One camera faced from the house toward the arena, the other from the far end of the garden toward the arena and house (Figure 4); views from different angles facilitated fox identification, which was based on a mixture of anatomical features such as scars, injuries, tail length, and coat patterns (Dorning & Harris, 2019d). In each territory, social status (dominant or subordinate) was established by entering each pair‐wise interaction over the 20 days of video recording into a scoring matrix (Kleiman, 1967; Schenkel, 1967; Vrolijk, 2011). Where possible we identified the dominant male and female in each group: All other foxes were classified as subordinates. Sex could not be determined for all subordinates, and we did not try to establish their rank order.

### 2.2 Neophobia and wariness stimuli

**FIGURE 5:**
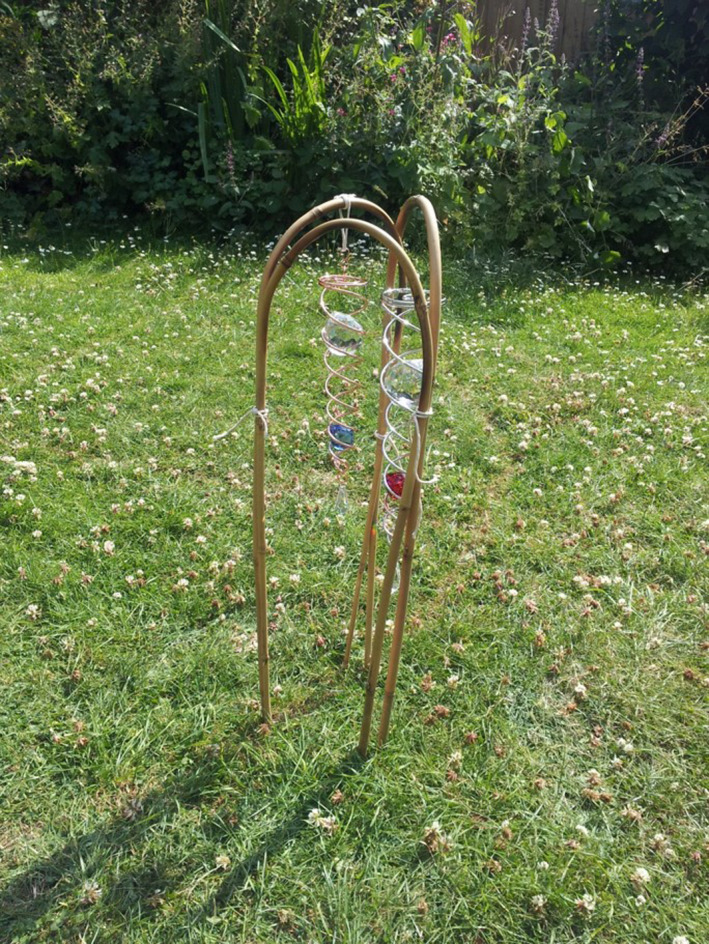
The object used for the neophobia test. The hanging ornaments spun in the wind and reflected light. DVDs were attached to the bamboo frame to increase reflectivity. The numerous sources of nocturnal light pollution (street lights, house lights, security lights, car lights, etc.) ensured that the neophobia stimulus was constantly changing day and night

A standard test for neophobia is to introduce novel objects to a familiar foraging patch (Mettler & Shivik, 2007; Moretti et al., 2015; Réale et al., 2007). We used a bamboo garden ornament with two reflective metallic ornamental baubles (Figure 5), and attached DVDs to the bamboo frame to increase reflectivity. The baubles and DVDs moved with the wind: There were numerous sources of nocturnal light pollution (street lights, house lights, security lights, car lights, etc.), so the neophobia stimulus was constantly changing day and night.

Wariness behavior is a response to a potentially threatening stimulus, such as predator odors, particularly those of predators with a long history of coevolution (Steindler et al., 2018; Valcarcel & Fernández‐Juricic, 2009). We used garden twine soaked in commercially available wolf urine (Wolf Urine Lure 32 oz., DeerBusters) because it was commercially available and wolves have a long history of coevolution with foxes (Wikenros et al., 2017). Wolves (*Canis lupus lupus*) are conspecific with domestic dogs (*Canis lupus familiaris*), the main predators of urban foxes (Harris, 1981; Harris & Smith, 1987), and so wolf urine would be perceived as threatening but not as a novel stimulus (Haswell et al., 2018). Preliminary experiments in Bristol showed that wolf urine elicited a strong wariness response by urban foxes: This included approaching the stimulus and then withdrawing, heightened vigilance behavior, and body postural changes characteristic of canids (Harris & Knowlton, 2001; Travaini et al., 2013).

**FIGURE 6:**
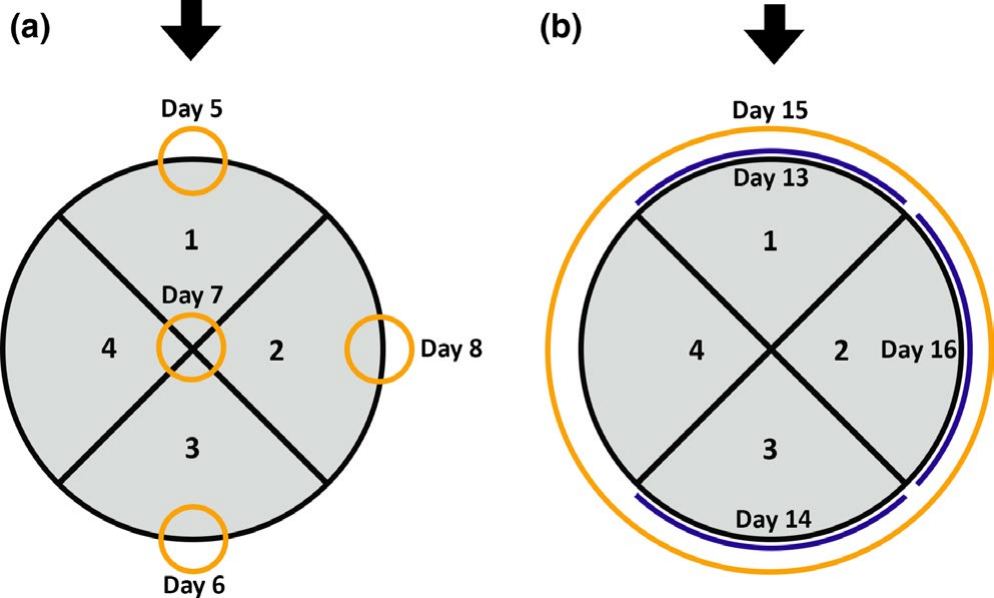
Positions of the two stimuli. (a) The orange circles indicate the position of the neophobia object on days 5 to 8. (b) The blue lines show the position of the wariness string on days 13, 14, and 16, and the orange circle shows the position of the wariness string on day 15. Quadrant 1 was closest to the entry point of the foxes (arrows), and quadrant 3 was closest to the house

Both experimental periods consisted of five stages, each 4 days long (Table 1); the positions of the neophobia and wariness objects on each day are shown in Figure 6.

**Table 1:** The five experimental stages

Test days	Experimental stage
1–4	*Pretreatment phase*: The foxes were allowed to forage in the circle without exposure to any stimulus to quantify their behavior prior to the tests
5–8	*Neophobia test*: The neophobia object was placed in a different position on each day so that the foxes would not become habituated to its location (Figure 6)
9–12	*Rest phase*: The foxes were allowed to forage in the circle without exposure to any stimulus and their behavior recorded prior to the wariness test
13–16	*Wariness test*: The wariness string was placed in a different position on each day so that the foxes would not become habituated to its location (Figure 6)
17–20	*Post‐treatment phase*: The foxes were allowed to forage in the circle without exposure to any stimulus and their behavior recorded after the wariness test

Note

Foxes were exposed to the neophobia object on days 5–8 and the wariness string on days 13–16. They were allowed to forage in the experimental area without any stimulus on days 1–4, 9–12, and 17–20.

### 2.3 Behavior scoring and analyses

Behavior was scored from the time food was placed in the middle of the circle to when all the food had been consumed. Time was measured to the nearest second from the moment a fox's nose crossed the boundary of the circle or quadrant to the moment its hind leg left the circle or quadrant. If a fox was standing in two quadrants at once, the time was recorded for both. The data were excluded from the analyses if a fox could not be identified reliably or if a fox entered the circle but did not feed. Foxes that did not feed were not included in the analyses because it was often unclear why they left without feeding: It could, for instance, have been a behavioral response to the stimulus or due to a disturbance in a nearby garden. An external circle of 4 m diameter (i.e., twice the diameter of the test circle) was drawn onto each video using the computer program Epic Pen (Tank Studios Ltd) to determine the outer hesitation and alarm behaviors.

We measured seven different behaviors relating to the fox's reactions to the experimental arena, neophobia object, and wariness string (Table 2). Outer hesitation, inner hesitation, outer alarm, and inner alarm represented hesitation and caution in the presence of the neophobia and wariness stimuli. Time per entry and frequency of entry represented avoidance, as a fox that was avoiding either stimulus was expected to spend less time in the circle or quadrant that contained the stimulus, and/or enter the circle or quadrant less often. We also recorded any behaviors directed at the neophobia or wariness object. Since the presence of conspecifics influences neophobia and wariness in a range of species (Fragaszy & Mason, 1978; Mainwaring et al., 2011; Moretti et al., 2015; Soma & Hasegawa, 2004; Visalberghi & Addessi, 2000; Webster et al., 2007), we also recorded the social context for each visit, that is, whether a dominant or subordinate was foraging at the same time as the focal fox, or whether the focal fox was alone.

**Table 2:** Description of the seven behaviors recorded after food had been placed in the middle of the experimental arena

Parameter	Description
Outer hesitation	Time taken from entering the external circle to reaching the boundary of the internal circle
Inner hesitation	Time taken from entering the internal circle, reaching the middle, and taking a piece of food
Outer alarm	Time spent alert and scanning the environment while in the external circle
Inner alarm	Time spent alert and scanning the environment while in the internal circle
Time per entry	Time spent in each quadrant/the circle as a whole per entry
Frequency of entry	The number of times a fox entered each quadrant/the circle as a whole per evening
Directed behavior	Any behavior directed at the stimulus, such as staring at, smelling, or pawing at the neophobia object or wariness string was recorded

All analyses were carried out in R (R Core Team, 2020; http://www.R‐project.org/) using RStudio (RStudio Team, 2020; https://rstudio.com/products/team/). Data from territories five and six in November/December were excluded from all analyses because it was not possible to identify individual foxes, and data from territory two in November/December and territories two and six in May were excluded because there were too few interactions to establish the social status of the foxes.

### 2.4 Testing for habituation

Since foxes were exposed to neophobia and wariness stimuli for four consecutive days during each test, it was possible that habituation decreased any behavioral responses as the experiment progressed. This could have affected our results if habituation occurred to a different extent in the two sampling periods (November/December and May) or in dominant and subordinate foxes.

To test whether this was the case, the neophobia and wariness datasets were analyzed separately using generalized linear mixed models (GLMMs), and likelihood ratio tests were used to identify the models that explained the most variation in the data. We used the data from test days 5, 6, and 8 (neophobia) and 13, 14, and 16 (wariness); days 7 and 15 were excluded because the neophobia object was in the middle of the circle above the food on day 7 and the wariness string surrounded the circle on day 15 (Figure 6), so any behavioral responses were not comparable to the other test days. Each model compared a fox's mean value for four behaviors (inner hesitation, inner alarm, outer hesitation, outer alarm) with season, social status, and neophobia or wariness test day. If test day was significant, we tested for an interaction with either social status or season. Fox identification was included as a random effect to account for repeated measures across test days and seasons.

Where behavioral variables had non‐normal distributions (neophobia and wariness inner and outer hesitation), the data were logged prior to inclusion in the model. Neophobia inner alarm contained some zeros (13/71 records) and so was transformed as log(1 + inner alarm). A large proportion of records were zeros for neophobia outer alarm (48/71), wariness inner alarm (31/89), and wariness outer alarm (67/89), and so, the data were converted to binary variables (i.e., some reaction or no reaction) and analyzed using a GLMM with binomial error.

### 2.5 Selecting the variables for analysis

Since we found no significant differences in behavior between the three nonexperimental periods (Table A1), we pooled them for comparison with the test values. We used the first six behaviors shown in Table 2 to calculate 17 variables for analysis (Table 3): “directed behavior” was excluded because interactions such as pawing and smelling the stimulus were infrequent and, while foxes often appeared to stare at the stimulus, it was difficult to distinguish this from general alertness, so these data were included in the calculations of DOA and EOA (see Table 3). Daily variables were calculated by subtracting the average total daily value of the pooled rest phases from the average total daily value during the neophobia or wariness tests. Per entry variables were calculated by subtracting the average value for each entry to the circle during the pooled rest phases from the average value for each entry during the neophobia or wariness tests. The variables that focused specifically on day 3 of the neophobia or wariness tests (when the neophobia object was in the middle of the circle and the wariness string completely surrounded the circle) were calculated by subtracting the average total daily or per entry value during the pooled rest phases from the total daily or average per entry value from day 3 of the neophobia or wariness tests.

**Table 3:** The calculated daily and per entry variables

Variable	Description of variable
DIH	Change in daily hesitation time while in the inner circle
DIA	Change in daily alarm time while in the inner circle
DOH	Change in daily hesitation time while in the outer circle
DOA	Change in daily alarm time while in the outer circle
DCT	Change in daily time spent in the circle
DTQT	Change in daily time spent in the test quadrant
DCT3	Change in total time spent in the circle on day three of the neophobia or wariness tests
FEC	Change in daily frequency of entry into the circle
FEC3	Change in frequency of entry into the circle on day three of the neophobia or wariness tests
FETQ	Change in daily frequency of entry into the test quadrant
EIH	Change in hesitation time per entry to the inner circle
EIA	Change in alarm time per entry to the inner circle
EOH	Change in hesitation time per entry to the outer circle
EOA	Change in alarm time per entry to the outer circle
ECT	Change in time spent in the circle per entry
ETQT	Change in time spent in the test quadrant per entry
ECT3	Change in time spent in the circle per entry on day three of the neophobia or wariness tests

Note

The test quadrant contained the neophobia object or had the wariness string bordering it. Variables included in the final analyses are shaded in green.

After examining the data, we decided to focus on per entry and frequency of entry variables for the analyses because they were less prone to extreme ranges than the variables for time spent in the quadrants or circle. The only exceptions to the exclusion of daily variables were DTQT and DCT3, which were used in preference to ETQT and ECT3 because some foxes never entered the test quadrant during the 4 days of either the neophobia or wariness tests, or never entered the circle on day three of the neophobia or wariness tests. While these animals had no value for ETQT and ECT3, there was still a value for DTQT and DCT3 because daily variables were calculated as zero minus the average daily rest value.

### 2.6 Principle components analysis

The analyses were based on 14 dominants and 20 subordinates across the two seasons (Table 4). Since the *SD* for many variables was large in comparison with the mean, we used a PCA to identify the underlying trends in the data as each variable provided a different measure of a fox's behavior when interacting with the neophobia or wariness stimulus (Jolliffe & Cadima, 2016; Nguyen & Holmes, 2019). To ensure that variables with larger variances did not have a disproportionate impact on the weights of the principal components, all variables were standardized to a mean of zero and a *SD* of one by subtracting the mean from each variable and dividing by the *SD* (Borgognone et al., 2001).

**Table 4:** Summary of the data collected over the two experimental periods

Season	November/December	May	Total
Total time recorded (hours)	2,880	2,880	5,760
Total time scored behaviorally (hours)	1,440	1,920	3,360
Number of dominant foxes	6	8	14
Number of subordinate foxes	11	9	20
Average number of visits to the circle per dominant fox	71	76	–
Average number of visits to the circle per subordinate fox	58	51	–
Average time per visit to the circle per dominant fox (seconds)	45	49	–
Average time per visit to the circle per subordinate fox (seconds)	41	43	–
Total visits to the circle	1,059	1,068	2,127
Total time foraging in the circle (hours)	12.5	14.0	26.5

The neophobia and wariness phases of the experiment used the same response measures (Table 3) because preliminary PCAs carried out separately on the neophobia and wariness data produced almost identical biplots, with the same relationship between PC1/PC2 and each of the response measures. So we combined the two datasets for the PCAs to capture the relationships between the different variables.

The first PCA used the total behavior dataset (Table 2) and contained two of each variable for most foxes in each season, that is, one for each for the neophobia and wariness tests. One fox did not enter the circle during the neophobia test in December, and so only provided a value for wariness behavior. The second PCA used the social context dataset and contained up to four values for each variable per fox in each season, that is, for a fox foraging alone and in the presence of others during both the neophobia and wariness tests. Not all foxes had all four variables as some were never observed foraging in the presence of others. Foraging with a dominant and a subordinate were combined as there were insufficient data for separate analyses.

### 2.7 Linear mixed models

Following the PCA analyses, linear mixed models were used to analyze the relationship between PC1 and social status, season, and social context. We used linear mixed models so that “fox” could be included as a random effect, thereby controlling for the 12 foxes present in both seasons; five were only present in December and five only in May. Although it was unlikely that foxes would remember the experimental set‐up after the 6‐month interval between test periods, linear mixed models accounted for any possible pseudoreplication. Territory number was also tested as a random effect to account for any influence of social group dynamics and foraging territory on the relationship between PC1 and the factors examined. Likelihood ratio tests were used to identify the models that explained the most variation in the data.

## 3 RESULTS

Across both seasons, we recorded 2,127 visits to the experimental arena (circle) in 5,760 hr; dominant foxes made more visits to the circle than subordinates, although average time per visit was similar (Table 4).

### 3.1 Habituation to the stimuli

We found no evidence that the foxes habituated to the experimental stimulus. For the neophobia stimulus, GLMMs and likelihood ratio tests showed that test day had no significant effect on inner (*X^2^* = 1.86, *df* = 2, *p* = .394) or outer hesitation (*X^2^* = 4.54, *df* = 2, *p* = .103). Inner alarm was significantly affected by test day (*X^2^* = 19.26, *df* = 2, *p* = 6.58e−05) but there was no significant interaction with social status (*X^2^* = 1.19, *df* = 2, *p* = .551) or season (*X^2^* = 1.23, *df* = 2, *p* = .541), that is, there was no difference in the level of habituation between dominants and subordinates, or across the two seasons. A Q–Q plot and residuals versus fitted values plot showed that a model testing the effect of social status, season, and neophobia test day on outer alarm was a poor fit, so we tested the significance of test day in two separate models with exclusion of either social status or season. Test day had no significant effect on outer alarm when tested alongside social status (*X^2^* = 5.04, *df* = 2, *p* = .080). Test day had a significant effect on outer alarm when tested alongside season (*X^2^* = 6.58, *df* = 2, *p* = .037), but there was no significant interaction with season (*X^2^* = 4.51, *df* = 2, *p* = .105).

For the wariness stimulus, test day had no significant effect on inner hesitation (*X^2^* = 2.28, *df* = 2, *p* = .320), inner alarm (*X^2^* = 0.12, *df* = 2, *p* = .941), outer hesitation (*X^2^* = 1.87, *df* = 2, *p* = .393), or outer alarm (*X^2^* = 4.58, *df* = 2, *p* = .102).

### 3.2 Influence of social status

**FIGURE 7:**
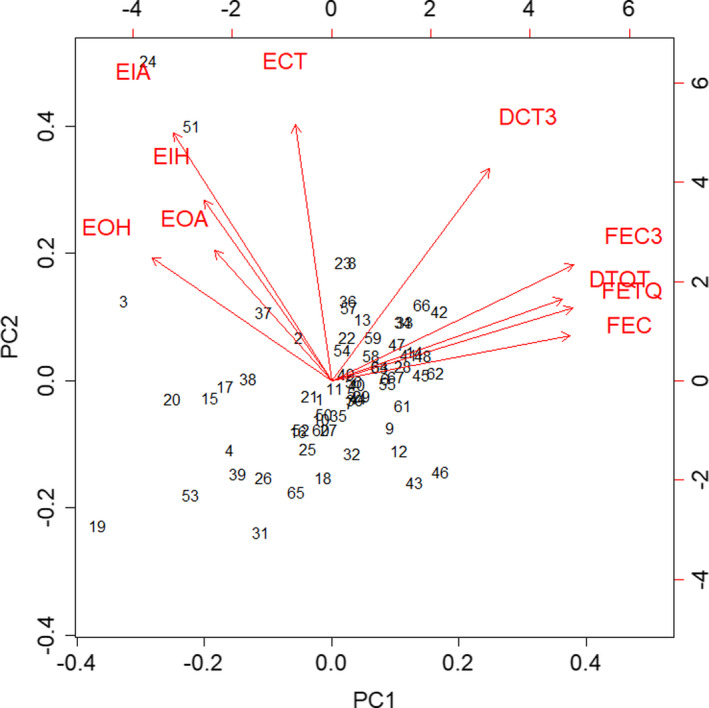
Biplot of the PCA for the total behavior dataset (neophobia and wariness data for each fox with no social context). The data points represent individual foxes. The arrows represent the relationship between PC1 and PC2 and each of the original variables; they show the direction, their length, and the strength, of the effect

**FIGURE 8:**
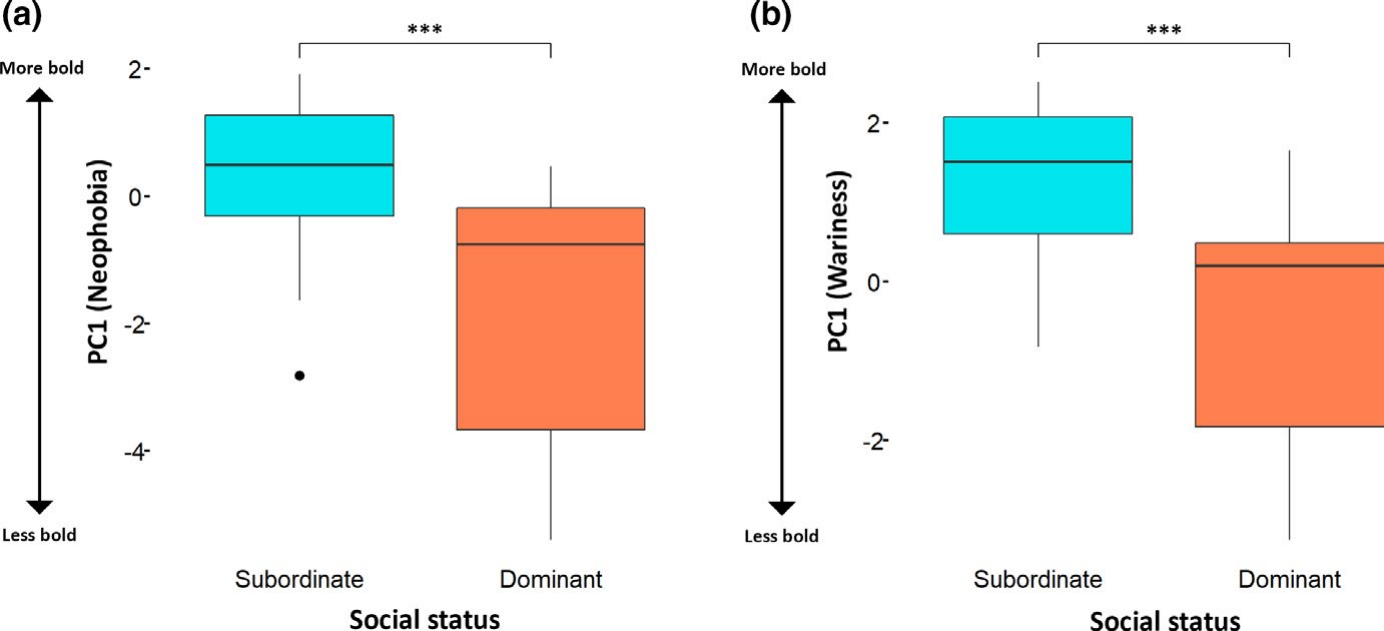
Boxplots showing the effect of social status on neophobia (a) and wariness (b). PC1 represented boldness, that is, foxes were more neophobic/wary as PC1 decreased. *** = likelihood ratio test *p* < .001 (Tables A2, A3 and A2, A3). The boxes show the median and upper and lower quartiles, and the whiskers extend one‐and‐a‐half times the interquartile distance from the upper and lower quartiles. The point denotes an outlier

In the PCA of the total behavior dataset, that is, neophobia and wariness data for each fox with no social context, PC1 had an *SD* of 1.79 and captured 32.2% of the variance. PC1 represented boldness in response to the two stimuli (Figure 7). As PC1 (boldness) decreased, foxes hesitated longer per entry to the outer (EOH) and inner circles (EIH), were alarmed for longer per entry to the outer (EOA) and inner circles (EIA), and spent less time in the circle per entry (ECT). As PC1 (boldness) increased, foxes entered the circle (FEC) and test quadrant (FETQ) more frequently, entered the circle more frequently on day three of the neophobia and wariness tests (FEC3), spent more time in the test quadrant (DTQT), and spent more time in the circle on day three of the neophobia and wariness tests (DCT3). PC2 had an *SD* of 1.58 and captured 25.0% of the variance, but was not used in further analyses because its biological significance could not be established. Having combined the datasets to produce a PC1 value for each fox, the neophobia and wariness datasets were analyzed separately using linear mixed models with individual fox as a random effect. Table A2 shows the results of the likelihood ratio tests, and Table A3 shows the best fitting model. Social status had a significant effect on PC1, that is, dominant foxes were more neophobic than subordinates (Figure 8). Season had no effect on neophobia (Table A2).

For wariness, we tested four linear mixed models, with individual fox as a random effect; Table A4 summarizes the results of the likelihood ratio tests, and Table A3 shows the best fitting model. Social status had a significant effect on PC1: Dominant foxes were more wary than subordinates (Figure 8). Season had no effect on wariness (Table A4).

### 3.3 Influence of social context

**FIGURE 9:**
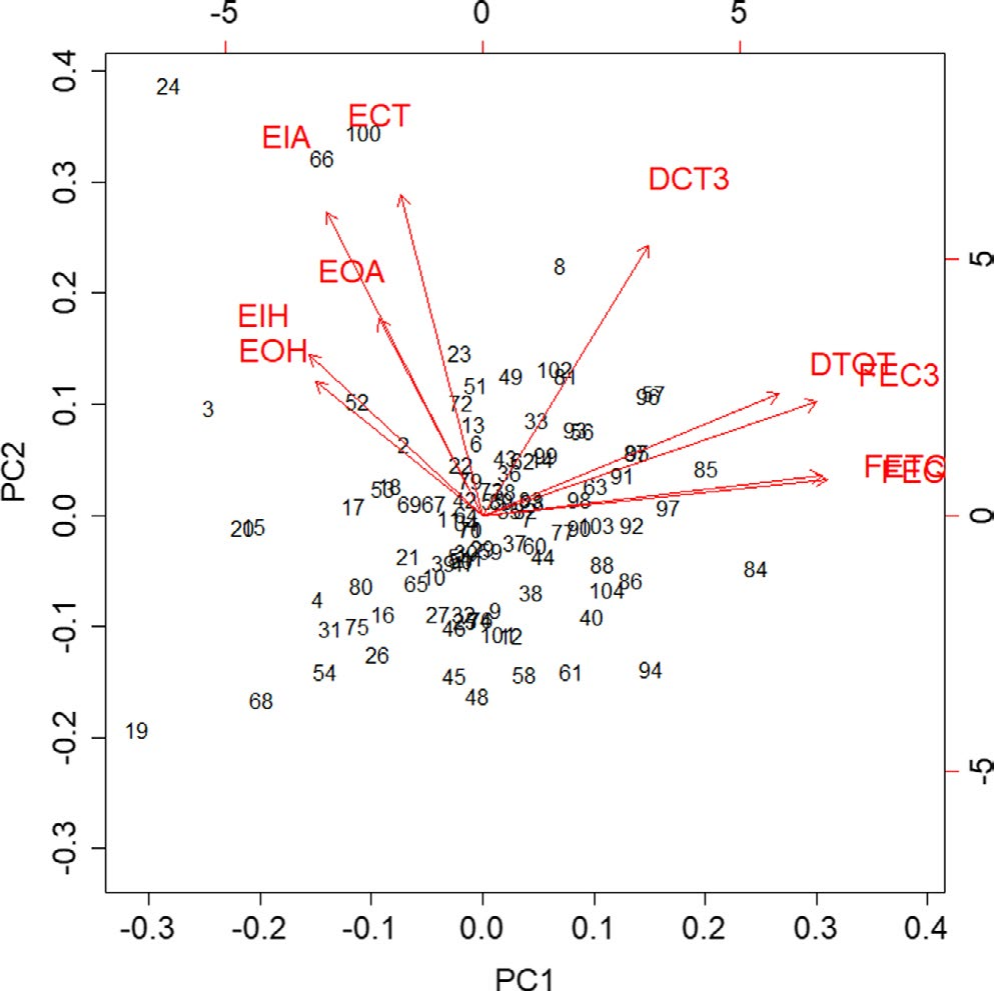
Biplot of the social context PCA. The social context data included four variables for most foxes in each season, one for a fox foraging alone during the neophobia test, one for a fox foraging with others during the neophobia test, one for a fox foraging alone during the wariness test, and one for a fox foraging with others during the wariness test. The data points represent individual foxes. The arrows represent the relationship between PC1 and PC2 and each of the original variables; they show the direction, their length, and the strength, of the effect

In November/December, foxes visited the arena alone on 886 occasions and 182 times in the presence of other foxes. In May, foxes visited alone on 861 occasions and 198 times in the presence of other foxes. We used a PCA on the social context dataset, that is, neophobia and wariness data for each individual with and without other foxes present. PC1 had an *SD* of 1.79 and captured 32.0% of the variance and represented boldness in response to the two stimuli (Figure 9). As PC1 (boldness) decreased, foxes hesitated more, displayed alarm for longer, and spent less time in the circle. As PC1 (boldness) increased, foxes entered the circle and test quadrant more frequently and spent more time in the circle and test quadrant. PC2 had an *SD* of 1.48 and captured 21.8% of the variance, but was not used in further analyses because its biological significance could not be established. Having combined the datasets to produce a PC1 value for each fox, the neophobia and wariness datasets were analyzed separately using linear mixed models.

All the linear mixed models used in the neophobia and wariness analyses contained individual fox as a random effect, and season was excluded as it had no effect on either behaviors (Section 3.2). Table A5 shows the results of the likelihood ratio tests on the neophobia models, Table A6 shows the results of the likelihood ratio tests on the wariness models, and Table A7 shows the best fitting models for each stimulus.

**FIGURE 10:**
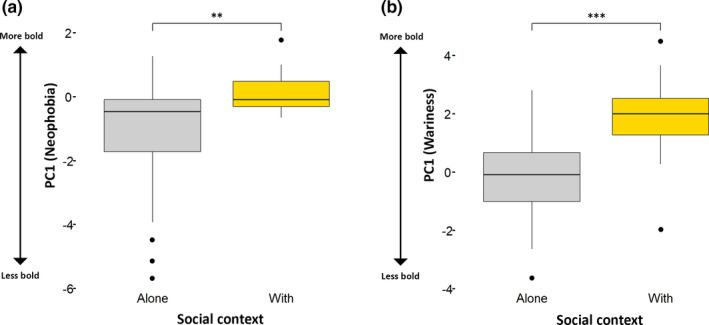
Boxplots showing the effect of social context, that is, whether foxes were alone when foraging (Alone) or another fox was present (With), on neophobia (a) and wariness (b). PC1 represented boldness, that is, foxes were more neophobic/wary as PC1 decreased. ** = likelihood ratio test *p* < .01, *** = *p* < .001 (Tables A5, A6 and A5, A6). The boxes show the median and upper and lower quartiles, and the whiskers extend one‐and‐a‐half times the interquartile distance from the upper and lower quartiles. The points denote outliers

Social status had a significant effect on PC1, that is, dominant foxes were more neophobic and more wary than subordinates. Social context also had a significant effect: Foxes foraging alone were more neophobic and more wary than when foraging in the presence of others (Figure 10). There was a significant interaction between social context and social status for neophobia (Table A5), with social context having less effect on subordinates. Subordinates were tested separately, and a likelihood ratio test comparing a model representing PC1 and its relationship with social context with a null model revealed a marginal but not statistically significant difference in the amount of variation explained (*X^2^* = 3.10, *df* = 1, *p* = .078). This suggests that subordinates foraging alone were slightly more neophobic than when foraging in the presence of others, although the effect was less pronounced than in dominants (*X^2^* = 8.19, *df* = 1, *p* = .004). There was no significant interaction between social status and social context for wariness (Table A6).

## 4 DISCUSSION

Quantified data on human–wildlife interactions help maximize societal benefits and minimize the potential for conflict (Alexander & Draper, 2019). Since animal personality is likely to influence human attitudes (both positive and negative) to urban foxes, it is surprising that it has not been considered hitherto, for example, Soulsbury and White (2019). We provide the first quantified analysis of how urban fox personality varies with social status and how a fox's personality might influence its interactions with people and their attitudes to urban foxes.

### 4.1 Boldness in urban foxes

While individual behaviors can be both a predictor and a consequence of social status in domestic fowl (*Gallus gallus domesticus*) (Favati et al., 2014), few studies have investigated the relationship between personality and dominance in groups of animals in the wild (Devost et al., 2016). Our data show that personality traits in urban foxes are linked to social status. Dominant foxes displayed heightened levels of both neophobia and wariness, whereas subordinates were more exploratory and more likely to take risks. So, for instance, while dominants and subordinates share food patches, dominant foxes visit more food patches within their territory, spend more time in predictable food patches, and feed earlier than subordinates. Subordinates forego foraging efficiency to mitigate intragroup competition and are more likely to visit food patches outside their territory, exposing themselves to increased sources of novelty and potentially threatening stimuli (Dorning & Harris, 2017). The exploratory and risk‐taking behavior of subordinate foxes probably also explains why they are easier to trap (Baker, Harris, et al., 2001), their extraterritorial mating strategies (Baker, Funk, Bruford, et al., 2004), and why they live significantly shorter lives than dominants (Baker et al., 1998).

Heightened levels of both neophobia and wariness in dominants appear to be common traits in canids. Alpha coyotes were less likely to be caught on camera traps than subordinates (Séquin et al., 2003); dominant coyotes tracked human activity through their territories, suggesting increased wariness (Séquin et al., 2003); dominant coyotes fed less than subordinates in the presence of a frightening sound and light stimulus (Darrow & Shivik, 2009); and dominant coyotes were less likely to be trapped and snared than subordinates (Sacks et al., 1999). However, the level of response may vary between species and populations. For instance, while culpeo foxes (*Pseudalopex culpaeus*) reduced visits to a bait station by 80% in the presence of a neophobia stimulus, South American grey foxes (*Pseudalopex griseus*) only reduced visitations by 10% when exposed to the same stimulus, possibly because they are more opportunistic predators (Travaini et al., 2013).

### 4.2 Seasonal and social effects on boldness

Contrary to expectations, we found no seasonal effects on neophobia or wariness. We expected that both subordinates and dominants would be less neophobic and less wary in May when provisioning cubs, as fox movements and mortality are both increased (Baker et al., 2007; Harris & Smith, 1987), and that this effect would be more pronounced in dominants, since they are primarily responsible for cub provisioning (Baker et al., 1998). The lack of significance in our data may be because season has a subtler effect on neophobia and wariness than social status, and so requires a larger dataset to detect a difference.

Foxes displayed higher levels of both neophobia and wariness when foraging alone; similar behavior has been recorded in a variety of species (Fragaszy & Mason, 1978; Moretti et al., 2015; Moscovice & Snowdon, 2006; Visalberghi & Addessi, 2000). However, dominant foxes were significantly less neophobic in the presence of others, whereas the effect was only marginally significant for subordinates, probably because subordinates were generally less neophobic than dominants. In contrast, both dominant and subordinate foxes were significantly less wary when foraging in the presence of others, probably because the wariness string represented a potential threat, whereas the neophobia object was simply something new in the environment.

### 4.3 Interactions between humans and urban foxes

Understanding the factors that influence personality traits in urban wildlife is fundamental to understanding human–wildlife interactions. Our data show that, despite frequent exposure to a diversity of sources of both novelty and potential danger in the urban environment, neophobia and wariness still influence urban fox behavior, with dominants more neophobic and wary than subordinates. While we were unable to establish the rank order of subordinates, it seems probable that particularly bold foxes are the lower ranking members of a social group. This would explain why, when the fox population in Bristol crashed to extremely low levels following an epizootic of sarcoptic mange (*Sarcoptes scabiei*) (Baker, Funk, Harris, et al., 2004; Baker et al., 2000), bold foxes were virtually nonexistent for the following decade, when fox social groups consisted predominantly of pairs with few or no subordinates (Baker, Newman, et al., 2001). Bold foxes only started to reappear in Bristol after social group sizes expanded to include subordinates, and they became more common as fox social group sizes increased (Harris, 2020).

In our study area, the high annual rate of retention of dominant status and the higher annual mortality rates of subordinates meant that the majority of subordinate foxes never became dominant (Baker et al., 1998). This is particularly important when considering any interventions that involve culling, which disrupts social group cohesion and encourages dispersing foxes to move into the culled area, leading to increased numbers (Baker & Harris, 2006; Dorning & Harris, 2019a). Increasing the number of less neophobic and less wary subordinates has the potential to enhance, not reduce, the number of human–urban fox interactions.

## 5 CONCLUSIONS

Whether or not an animal is viewed as a nuisance can have a significant impact on wildlife management (Barrett et al., 2019). At present, British perceptions of urban foxes are influenced by media reports, which are typically sensational and negative (Bridge & Harris, 2020; Cassidy & Mills, 2012). Nevertheless, it is important to understand human–fox interactions to foster coexistence in urban areas, especially since attitudes vary and one in seven British adults actively encourage foxes to visit their garden, generally by providing food (Baker, Funk, Harris, et al., 2004; Guthrie, 2019). Currently, there are few data on how, why, and/or when humans and urban foxes interact. While it has been suggested that living in urban areas could select for bolder or more cognitively advanced individuals that habituate to humans (Barrett et al., 2019; Scott, 2017), similar human–fox interactions have been recorded in rural fox populations, especially in protected areas where they are not persecuted, for example, Tsukada (1997). We found that social status influenced neophobia and weariness in foxes: since group size (and hence the proportion of subordinates in a population) is likely to vary between habitats, our data highlight the need to include social status in analyses that compare personality traits in different populations or habitats (c.f. Breck et al., 2019).

We were unable to investigate all the factors that might influence a fox's personality, and future work should consider the effects of an animal's health on human–urban fox interactions (Koski, 2014). Disease, particularly the effects of novel parasites such as *Toxoplasma gondii*, can induce risk‐taking and other behaviors that influence an animal's social interactions with conspecifics, other species, and people (Johnson et al., 2018; Poulin, 2018).

## CONFLICT OF INTEREST

The authors declare that they have no conflict of interest.

## AUTHOR CONTRIBUTIONS

**Roberto Padovani:** Conceptualization (equal); data curation (lead); formal analysis (lead); funding acquisition (equal); investigation (lead); methodology (equal); project administration (lead); resources (equal); software (lead); supervision (equal); validation (lead); visualization (lead); writing‐original draft (equal); writing‐review & editing (equal). **Zhuoyu Shi:** Conceptualization (supporting); methodology (supporting); writing‐original draft (supporting); writing‐review & editing (supporting). **Stephen Harris:** Conceptualization (equal); data curation (supporting); formal analysis (supporting); funding acquisition (equal); investigation (supporting); methodology (equal); project administration (supporting); resources (supporting); software (supporting); supervision (lead); validation (supporting); visualization (supporting); writing‐original draft (equal); writing‐review & editing (lead).

## ETHICAL APPROVAL

The study was observational, and no animals were caught or handled. The study was approved by the University of Bristol Animal Welfare and Ethics Committee (UB/14/015).

## Data Availability

The datasets generated and analyzed during the current study are available from the Dryad digital repository: https://datadryad.org/stash/dataset/doi:10.5061/dryad.np5hqbzrb.

## Appendix A

**Table A1:** Results of the Friedman tests comparing fox behaviour during the pre‐treatment, rest and post‐treatment phases for dominant and subordinate foxes in November/December and May

Behavioural variable	Social status	Season	Test results
Inner hesitation	Dominant	November/December	*X^2^* = 1.333, *p* = .513
		May	*X^2^* = 1.750, *p* = .417
	Subordinate	November/December	*X^2^* = 5.250, *p* = .072
		May	*X^2^* = 0.222, *p* = .895
Inner alarm	Dominant	November/December	*X^2^* = 2.333, *p* = .311
		May	*X^2^* = 0.250, *p* = .883
	Subordinate	November/December	*X^2^* = 0.250, *p* = .883
		May	*X^2^* = 1.556, *p* = .459
Outer hesitation	Dominant	November/December	*X^2^* = 1.130, *p* = .568
		May	*X^2^* = 1.750, *p* = .417
	Subordinate	November/December	*X^2^* = 3.000, *p* = .223
		May	*X^2^* = 1.118, *p* = .572
Outer alarm	Dominant	November/December	*X^2^* = 1.909, *p* = .385
		May	*X^2^* = 0.133, *p* = .936
	Subordinate	November/December	*X^2^* = 2.467, *p* = .291
		May	*X^2^* = 1.444, *p* = .486

Note

None of the results were significant, i.e. fox behaviour was consistent during the three non‐experimental phases. Friedman tests were used because the data were not normally distributed. The behavioural variables are defined in Table 2; all *df* = 2.

**Table A2:** The results of the likelihood ratio tests comparing different models for neophobia using the total behaviour neophobia data set

Likelihood ratio test	Neophobia models	AICc	Test results	Interpretation
1	PC1 ~ social status + season	127.944	*χ^2^* = 1.582 *p* = .209	No significant interaction between social status and season
	PC1 ~ social status + season + social status * season	129.370		
2	PC1 ~ social status	126.283	*χ^2^* = 1.134 *p* = .287	Season does not significantly improve the model
	PC1 ~ social status + season	127.944		
3	PC1 ~ season	139.037	*χ^2^* = 13.887 *p* = 1.941e−04	Social status significantly improves the model
	PC1 ~ social status + season	127.944		
4	PC1 ~ 1	139.315	*χ^2^* = 15.632 *p* = 7.692e−05	Confirms the effect of social status on PC1
	PC1 ~ social status	126.283		

Note

The total behaviour dataset made no distinction between foxes foraging alone and those foraging when other foxes were present. All models included fox identification as a random effect; all *df* = 1.

**Table A3:** The equations and outputs of the best fitting models for neophobia and wariness using the total behaviour neophobia and wariness datasets

Behaviour	Final model	Fixed effect	Estimate	*SE*	*t*
Neophobia	PC1 ~ social status + (1 | fox name)	Intercept	0.482	0.401	1.200
		Social status	−2.514	0.566	−4.442
Wariness	PC1 ~ social status + (1 | fox name)	Intercept	1.274	0.286	4.458
		Social status	−1.788	0.445	−4.016

Note

These datasets made no distinction between foxes foraging alone and those foraging when other foxes were present.

**Table A4:** The results of the likelihood ratio tests comparing different models for wariness using the total behaviour wariness data set

Likelihood ratio test	Wariness models	AICc	Test results	Interpretation
1	PC1 ~ social status + season	123.921	*χ^2^* = 1.897 *p* = 1.684	No significant interaction between social status and season
	PC1 ~ social status + season + social status * season	124.992		
2	PC1 ~ social status	121.508	*χ^2^* = 0.350 *p* = .554	Season does not significantly improve the model
	PC1 ~ social status + season	123.921		
3	PC1 ~ season	134.122	*χ^2^* = 12.964 *p* = 3.175e−04	Social status significantly improves the model
	PC1 ~ social status + season	123.921		
4	PC1 ~ 1	132.603	*χ^2^* = 13.675 *p* = 2.174e−04	Confirms the effect of social status on PC1
	PC1 ~ social status	121.508		

Note

This dataset made no distinction between foxes foraging alone and those foraging when other foxes were present. All models included fox identification as a random effect; all *df* = 1.

**Table A5:** The results of likelihood ratio tests comparing different models for neophobia from the social context neophobia dataset

Likelihood ratio test	Neophobia models	AICc	Test results	Interpretation
1	PC1 ~ social status + social context	174.327	*χ^2^* = 5.60 *p* = .018	Significant interaction between social context and social status
	PC1 ~ social status + social context + social status * social context	171.346		
2	PC1 ~ social status + social context	174.327	*χ^2^* = 10.690 *p* = .001	Social status significantly improves the model
	PC1 ~ social context	182.519		
3	PC1 ~ social status + social context	174.327	*χ^2^* = 8.981 *p* = .003	Social context significantly improves the model
	PC1 ~ social status	180.810		
4	PC1 ~ 1	188.917	*χ^2^* = 10.492 *p* = .001	Confirms the effect of social status on PC1
	PC1 ~ social status	180.810		
5	PC1 ~ 1	188.917	*χ^2^* = 8.783 *p* = .003	Confirms the effect of social context on PC1
	PC1 ~ social context	182.519		

Note

The social context dataset distinguished between foxes foraging alone and those foraging when other foxes were present. All models included fox identification as a random effect; all *df* = 1.

**Table A6:** The results of likelihood ratio tests comparing different models for wariness from the social context wariness dataset

Likelihood ratio test	Wariness models	AICc	Test results	Interpretation
1	PC1 ~ social status + social context	192.828	*χ^2^* = 1.603 *p* = .205	No significant interaction between social status and social context
	PC1 ~ social status + social context + social status * social context	193.739		
2	PC1 ~ social status + social context	192.828	*χ^2^* = 12.827 *p* = 3.416e−04	Social status significantly improves the model
	PC1 ~ social context	203.239		
3	PC1 ~ social status + social context	192.828	*χ^2^* = 28.595 *p* = 8.920e−08	Social context significantly improves the model
	PC1 ~ social status	219.007		
4	PC1 ~ 1	225.423	*χ^2^* = 8.739 *p* = .003	Confirms the effect of social status on PC1
	PC1 ~ social status	219.007		
5	PC1 ~ 1	225.423	*χ^2^* = 24.507 *p* = 7.404e−07	Confirms the effect of social context on PC1
	PC1 ~ social context	203.239		

Note

The social context dataset distinguished between foxes foraging alone and those foraging when other foxes were present. All models included fox identification as a random effect; all *df* = 1.

**Table A7:** The equations and outputs of the best fitting models for neophobia and wariness from the social context neophobia and wariness datasets

Behaviour	Final model	Fixed effect	Estimate	*SE*	*t*
Neophobia	PC1 ~ social status + social context + social status * social context + (1 | fox name)	Intercept	−0.316	0.325	−0.971
		Social status	−2.005	04.96	−4.044
		Social context	0.651	0.448	1.453
		Social status * social context	1.640	0.756	2.170
Wariness	PC1 ~ social status + social context + (1 | fox name)	Intercept	0.338	0.252	1.343
		Social status	−1.302	0.334	−3.895
		Social context	2.064	0.337	6.131

Note

The social context dataset distinguished between foxes foraging alone and those foraging when other foxes were present.
